# Bilateral dorsal perilunate dislocation of wrist

**DOI:** 10.4103/0019-5413.61724

**Published:** 2010

**Authors:** P Ranga Chari

**Affiliations:** Department of Orthopaedics, Osmania Medical College, Hyderabad - 500 001, India

**Keywords:** Bilateral dorsal perilunate dislocation, wrist, mechanism

## Abstract

We present a case of simultaneous dorsal perilunate dislocation of both wrists, without a history of fall on outstretched hands. In contrast, it appeared that the mechanism was reverse. His hands were held in radial deviation with wrists in full palmar flexion. The forearms were in neutral position and elbows in mid-flexion. The wrists were suddenly and forcibly pronated. The radiographs of both wrists showed dorsal perilunate dislocation with avulsion fracture of the tip of ulnar styloid process and avulsion fracture of posterior horn of lunate. Radial translation of the carpal bones was also noted. The mechanism is proposed and discussed.

## INTRODUCTION

Fractures or dislocations of carpal bones usually result from a fall on outstretched hands with wrists in hyperextension. This usually happens in motor vehicle accidents. Dorsal perilunate dislocation of wrist is one of the commonest patterns.^1^,^2^ Some authors include isolated anterior dislocation of lunate under posterior perilunate dislocation.^3^

The present concept of the mechanism of dorsal perilunate dislocation in literature is a fall on outstretched hand causing dorsiflexion and axial impaction of the carpal bones on the forearm bones with ulnar deviation and supination of the wrist over the fixed pronated forearm.^3^ Intercarpal supination seems to be a main factor responsible to determine the nature of the capsuloligamentous lesions resulting in posterior perilunate dislocation.^3^ During the ulnar deviation, the head of the capitate tends to drive the scaphoid outward and to separate the later from the lunate. A violent blow following the fall on hyperextended hand ruptures the scapholunate connections. The head of the capitate then slips between the scaphoid and the lunate and dislocates posteriorly dragging the carpus with it.

## CASE REPORT

**Figure 1:**
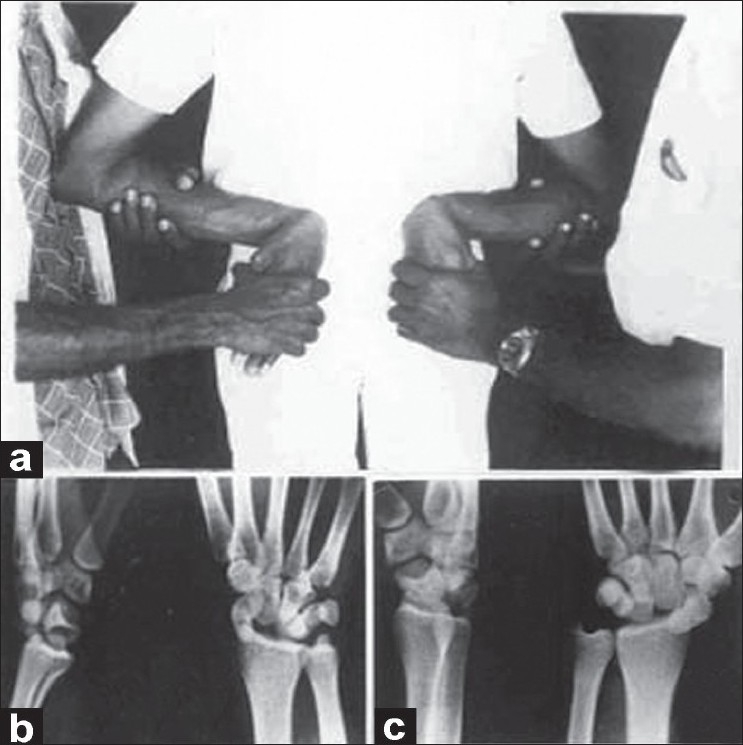
(a) Clinical photograph showing the position of limbs held on back in forcible full volar flexion, radial deviation and pronation at the wrists.(b) Lateral and anteroposterior X-rays of left and right.(c) wrists showing posterior perilunate dislocation. Note: Irregularity of posterior horn of lunate in lateral view

**Figure 2:**
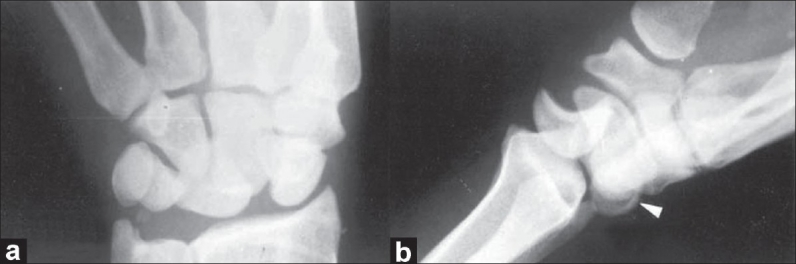
(a) Anteroposterior skiagram of wrist with posterior perilunate dislocation showing avulsion fracture of the tip of ulnar styloid process.(b) Lateral view of wrist with posterior perilunate dislocation showing avulsion fracture defect in posterior horn of lunate. Note: Arrow head is pointing at the fracture fragment

**Figure 3:**
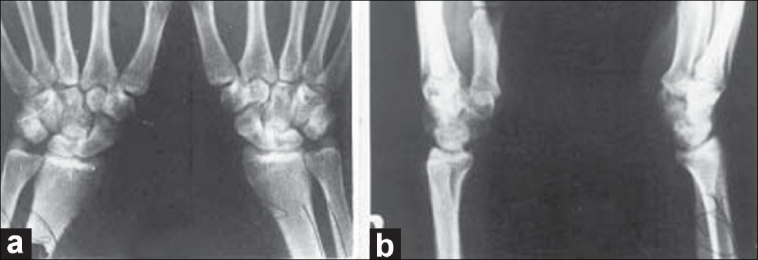
Two months post reduction skiagram (a) anteroposterior (b) lateral views of both wrists showing maintained reduction

A 25-year-old man, an apprentice welder in a heavy steel industry, was returning home after watching a late-night movie show. When he was walking alone, before he realized two robbers followed and stopped him for money. Then the two robbers one on each side twisted his arms toward his back. They held his forearms in neutral and elbows in 90° flexion. His wrists were forced in full volar flexion, radial deviation, and pronation. The situation was simulated in the diagram [Figure 1a]. While he was struggling to free himself, both the intruders were forcibly pronating his hands with the wrists kept in full volar flexion. He cried loudly because of severe shooting pain in hands and wrists. The intruders escaped from the scene shortly. His hands and wrists were swollen and very painful. Radiographs were obtained on the next day, and it revealed dorsal perilunate dislocation of both wrists [Figure 1b and c]. Avulsion fractures of the posterior horn of the lunates, radial translation of the carpal bones and fracture of the tips of ulnar styloid processes were noticed [Figure 2a and b]. The patient was treated by closed reduction under general anesthesia. Gentle traction was applied to the hand in slight volar flexion over the steadily held forearm in neutral position on both sides. The wrists, with traction on, were gently deviated radially and pronated. Then, the hands were supinated with continued traction while pushing the dorsally displaced carpi volarward. The dislocations got reduced without much difficulty [Figure 3]. A well-padded, below-elbow POP back slab was applied, keeping the wrist in 15°-20° volar flexion and 20°-25° ulnar deviation on both sides. After four weeks of immobilization, active exercises were started. The wrists regained almost full range of movements in about 10 weeks time. The patient was lost to further follow-up.

## DISCUSSION

For the first time, Mouchet^4^ thought the initial trauma to be sudden hyperextension of the wrist. This concept was revived and extended by Wagner.^1^ In his opinion, at the time of hyperextension of the wrist associated with ulnar deviation of the hand, the posterior border of the radius offers resistance to the capital head and tears the scapulolunate ligament, resulting in classical posterior perilunate dislocation.

In the present case, the mechanism of posterior perilunate dislocation is contrary to what is being taught and described in books. The hands in this case, held in radial deviation with wrist in full volar flexion, were forcibly pronated over steadily held forearms in neutral position. Because of this maneuver, the ulnar collateral ligament becomes tight and dorsal intercarpal ligaments with capsule get stretched. On the volar aspect of the wrist, the tough radioscaphocapitate and radiolunate ligaments along with other intercarpal ligaments with capsule become lax. If the hand in such a position is forcibly pronated, it results in the avulsion of ulnar collateral ligament of the wrist with or without fracture of the tip of ulnar styloid process. Further pronation of the hand causes rotation of the head of the capitate in lunate producing avulsion of the dorsal lunocapitate with avulsion fracture of the posterior lip or horn of lunate. Finally, the same pronation force produces tear of the scapholunate ligaments, which results in the posterior dislocation of the carpi to lie on the posterior surface of the lunate and distal articular margin of the radius.

Posterior perilunate dislocation of the wrist could also happen if the individual falls forwards on the dorsum of the radially deviated hand with wrist in full volar flexion and on outstretched opposite hand, to result in forcible pronation twist of the wrist with hand in radial deviation.

Unrelated to the trauma, a persistent bilateral posterior perilunate dislocation due to generalized laxity in case of Marfan syndrome was reported.^5^ But, in eight other Marfan patients, carpal instability could not be demonstrated in skiagrams of the wrists. In such cases to demonstrate unsuspected carpal instability, stress skiagrams of wrists are advised.^5^ Our patient had no evidence of generalized ligament laxity as in Ehler Danlos or Marfan syndrome.
